# A school-level randomized preliminary study of in-classroom physical activity breaks among fifth graders

**DOI:** 10.1016/j.jesf.2026.200498

**Published:** 2026-07-13

**Authors:** Yanping Duan, D.L.I.H.K. Peiris, Corneel Vandelanotte, Wei Liang

**Affiliations:** a Department of Sports and Health Sciences, Hong Kong Baptist University, Hong Kong Special Administrative Region of China; b Department of Sport Science and Physical Education, University of Kelaniya, Sri Lanka; c Physical Activity Research Group, Central Queensland University, Australia; d School of Physical Education, Shenzhen University, China

**Keywords:** Accelerometers, BCT, COM-B model, Seated learning, Pre-adolescents

## Abstract

**Background:**

In-classroom physical activity breaks (IcPABs) are a pragmatic strategy to reduce sedentary time and promote movement during instructional lessons. However, evidence from primary schools operating within a high-stakes examination-focused education system, where academic achievement in national assessments is prioritized in classroom instruction is limited from low- and middle-income countries. This study aimed to examine the preliminary effects and feasibility of a teacher-led, in-classroom physical activity break intervention on school-time movement behaviors, academic performance, and selected health-related outcomes among Sri Lankan fifth-grade students.

**Methods:**

220 students from two government primary schools in Sri Lanka (mean _age_ = 9.48 years ± 0.5, 61% male) were randomly assigned to (1) an intervention group (n = 100): attending thrice-a-day, 5-min IcPAB for 12 weeks; and (2) a control group (n = 120). Pre- and post-intervention assessments were conducted for academic achievement (standardized tests), movement behaviors (ActiGraph Gt3X), body-mass-index (anthropometric data), aerobic fitness (20m shuttle run test), and perceived stress (PSQ8-11). Generalized Linear Mixed Models were used for statistical analyses at exploratory level. Feasibility was evaluated using teacher logs, classroom observations and process evaluation records.

**Results:**

Significant group-by-time effects were observed for reading performance (82.2 ± 13.9 to 88.0 ± 12.9 vs 76.7 ± 10.5 to 77.6 ± 13.0 in controls), MVPA (27.2 ± 9.4 to 44.3 ± 9.9 vs 31.1 ± 8.4 to 24.3 ± 9.4), step count (5609 ± 1019 to 7769 ± 1339 vs 7998 ± 1023 to 3942 ± 1046), and sedentary school time (1490.7 ± 53.1 to 1475.4 ± 59.4 vs 1485.0 ± 22.9 to 1655.1 ± 40.8). The study fidelity assessment confirmed 1 to 4 times daily activity implementation. The process evaluation indicated satisfactory participant engagement and smooth implementation, suggesting the study was conducted as intended.

**Conclusion:**

This school level randomized preliminary study observed improvements in reading performance, while effects on mathematics and health-related outcomes were limited. However, in-classroom physical activity breaks were feasible to implement in an academically intensive Sri Lankan primary school context and were associated with favorable changes in school-time movement behaviors. Yet larger multi-school trials are expected to confirm these findings.

## 1 Introduction

Physical activity (PA) is recognized to provide numerous benefits for children, including elementary schoolers.^1^^,^^2^ PA at varying intensities can enhance academic achievement in subjects such as reading and mathematics,^3^^,^^4^ promote active learning through movements ranging from light to vigorous,^5^^,^^6^ and improve health indicators such as body-mass-index (BMI),^7^^,^^8^ aerobic fitness,^9^^,^^10^ and stress/test anxiety levels. 11, 12, 13 Despite these benefits, studies indicate that children rarely meet the recommended daily PA guidelines, such as engaging in at least 60 min of moderate-to-vigorous physical activity (MVPA)^14^^,^^15^ or consistently break up prolonged sitting periods. These guidelines are often unmet due to extended seated learning sessions required by demanding primary school curricula.^16^^,^^17^

There has been growing interest as a pragmatic strategy in incorporating brief, PA breaks into classroom settings.^15^ These in-classroom physical activity breaks (IcPAB) aim to help students accumulate sufficient PA without disrupting educational activities^18^ and to optimize their health and learning. However, there remains no consensus on the optimal duration and frequency of PA breaks. Many researchers suggest that while extended PA breaks are beneficial, they may be challenging for teachers to manage amidst their other responsibilities.^19^ Therefore, recent recommendations based on teacher feedback advocate for shorter PA breaks, such as 5-min sessions, that fit seamlessly within the classroom environment and support learning goals.^19^^,^^20^ Despite these recommendations, limited evidence exists on the effectiveness of 5-min IcPAB, particularly regarding their impact on academic achievement, movement behaviors, reduction in sedentary time, and health indicators.^18^

Importantly, the majority of IcPAB research has been conducted in high-income countries,^18^ where school infrastructure, teacher training, class sizes, and curricular flexibility differ substantially from those in low- and middle-income countries. As a result, the transferability of findings from these settings to low- and middle-income countries education systems remain uncertain. In countries such as Sri Lanka, primary education is highly examination-oriented, with Grade Five students preparing for a nationally competitive scholarship examination that significantly shapes classroom practices, instructional time allocation, and teacher priorities.^13^^,^^20^ This examination places particular emphasis on reading and mathematics performance, which are considered core determinants of academic success at this level. As a result, students often engage in extended seated learning sessions as teachers prioritise instructional time to cover both the formal curriculum and additional examination preparation. Findings from qualitative interviews with primary school teachers further confirm that reading and mathematics are perceived as priority academic outcomes within this context^20^ and prolonged seating time cause health concerns such as increased body-mass-index, lower cardiorespiratory fitness, and heightened stress.^13^^,^^20^

Given this strong academic emphasis, interventions implemented in such settings must demonstrate not only health-related benefits but also relevance to key academic outcomes valued by schools. Therefore, reading and mathematics achievement were selected as primary outcomes in the present study. This decision is further supported by existing literature demonstrating positive associations between physical activity and academic performance in these domains.^18^

However, beyond contextual differences, existing IcPAB studies have frequently focused on outcome effectiveness, with comparatively less attention paid to implementation processes, teacher fidelity, and feasibility in real-world classroom settings.^18^ This limitation is also evident in the broader physically active learning literature, where studies on physically active lessons have highlighted similar challenges related to teacher feasibility, classroom management, and implementation fidelity in real-world school environments. For example, recent research on physically active lessons has demonstrated that while such approaches are feasible and acceptable in primary school settings, their successful implementation depends heavily on teacher support, contextual adaptability, and integration into routine instructional practices.^21^ Behaviour change frameworks such as the Capability–Opportunity–Motivation–Behaviour (COM-B) model^18^ offer a useful lens for designing classroom-based interventions that are responsive to teacher constraints and student needs. The COM-B model proposes that behaviour occurs through the interaction of three key components such as capability (physical and psychological ability to perform a behaviour), opportunity (environmental and social factors that enable the behaviour), and motivation (reflective and automatic processes that direct behaviour).^22^ This model has been widely used to understand COM-B factors that might hinder or encourage an introduced behavior 23, 24, 25, so that researchers may design and implement behaviour change interventions by addressing those COM-B by treating those as barriers and facilitators influencing behavioural adoption. In the context of school-based physical activity interventions, the COM-B model provides a useful framework for understanding teachers' and students’ ability, motivation, and opportunities to engage in classroom-based physical activity despite curricular and time-related constraints.^20^ However, empirical evidence on COM-B–informed IcPABs in low- and middle-income country school settings remain scarce. In response, based on the COM-B model, an evidence-based, theoretically grounded 5-min IcPAB intervention was introduced for fifth-grade students in Sri Lanka, guided by findings from recent meta-reviews.^18^ The intervention primarily aimed at enhancing academic achievement, focusing on reading and mathematics while targeting movement behaviors such as PA levels, steps count, sedentary behavior, and health outcomes such as body-mass-index, aerobic fitness, and perceived stress. The findings expected to examine the preliminary effects and feasibility of a teacher-led, COM-B–informed in-classroom physical activity break intervention among Sri Lankan fifth-grade students, using a school-level randomized preliminary study design.

## 2 Materials and methods

Study protocol was published^13^ and registered with the ISRCTN registry (Ref: ISRCTN52180050). Ethics Review Committee of the University of Kelaniya, Sri Lanka (Ref: UOK/ERC/SS/2022/009) and Hong Kong Baptist University (Ref: SOSC-SPEH-2022-23_113) granted the ethical approval.

### 2.1 Trial design and participants

This study was conducted as a school-level randomized preliminary study with a parallel-group design with two government schools in Bandarawela Education Zone, Uva Province, Sri Lanka, from July to October 2022. Results followed CONSORT guidelines (Supplementary file 1). Details of the participants' enrollment strategy are published elsewhere.^13^ Briefly, five schools were invited to participate through convenience sampling, and two out of five consented to join the study. Within these schools, 270 students from seven parallel classes were recruited.

All Grade 5 students enrolled in the selected schools were eligible to participate. Exclusion criteria included medical conditions that contraindicated participation in light-to-moderate physical activity or inability to engage in classroom-based movement activities as advised by school records or parental notification.

A minimum of 198 participants were needed in both the intervention (IG) and control groups (CG) to detect an effect size of 0.21 ^13^ on academic achievement (mathematics), accounting for a 10% dropout rate. Given the small number of participating schools, the study was designed to examine feasibility and preliminary effects, rather than to provide confirmatory causal inference typical of a fully powered cluster-randomized controlled trial.

The targeted population was exposed to 6 h of regular classroom time (7.30 a.m. to 1.30 p.m.), including a 20-min lunch break according to the government primary school norms in Sri Lanka. In the government primary school setting, the teacher in charge of a class should teach all subjects in the class syllabus, such as mother tongue, mathematics, religion, environment, and English. Depending on the schools' interests, the teachers conducted additional classes after regular school hours to prepare students for the year-end national scholarship examination. Therefore, the study was designed to be implemented only during the standard classroom time for 12 weeks.

### 2.2 Randomization and blinding

Randomization occurred before baseline assessments. The two participating schools were randomly assigned at the school level, with one school allocated to the intervention group and the other to the control group. This approach ensured that all students within a school received the same treatment, preventing contamination between groups. A person blinded to the intervention content shuffled sealed envelopes with treatment allocations. Due to the nature of the intervention, blinding of teachers and students regarding the study's intentions was not possible. However, the teachers and students were unaware of the study's specific hypotheses.

### 2.3 The intervention

**Fig. 1:**
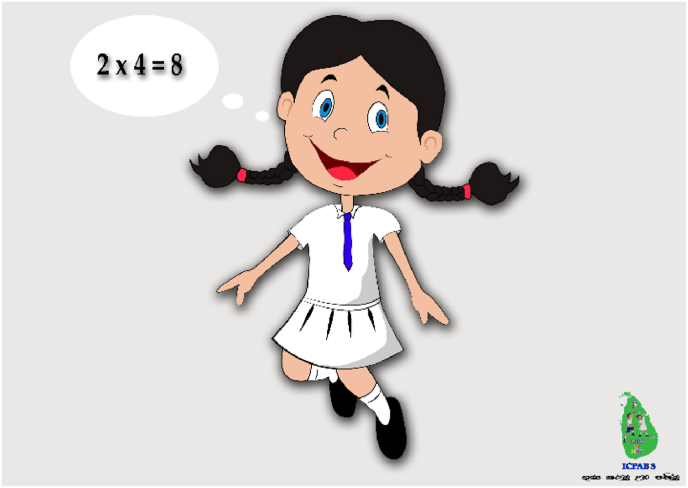
An IcPAB card used for arithmetic.

The IcPAB intervention was developed using seven steps,^13^ which explained in-depth in the study protocol paper: This process included a systematic review of existing RCT-based IcPAB interventions, qualitative interviews with primary school teachers in Sri Lanka, and the application of the COM-B model and Behaviour Change Techniques (BCTs) to design a contextually appropriate intervention. Teachers received intervention materials, including the manual, 20 IcPAB cards for teaching language-based reading and mathematics, timers, and a logbook, all written in Sinhala with English terms used where necessary. Each card had a picture resembling a particular IcPAB with written instructions on performing the activities, in addition to the instructions on its back. For example, in one mathematics-integrated IcPAB activity (Fig. 1) called “Let's Multiply and Jump”, students first performed guided breathing and rhythmic body movements. The teacher then presented multiplication problems, and students solved the arithmetic task mentally or on paper before demonstrating the numerical answer through the corresponding number of spot jumps. Teachers subsequently reviewed the correct answer on the board and provided positive reinforcement before returning to the lesson. Each activity session lasted approximately 5 min. The detailed manual of 20 IcPAB cards can be found elsewhere.^13^

Teachers in the IG were instructed to implement IcPAB for 5 min at least three times daily for 12 weeks. The cards aimed to reduce seated learning time without disrupting academic lessons, improve students' BMI, aerobic fitness, and reduce stress.^13^ Teachers were allowed to modify the IcPAB and document the changes in the logbook, with the principal investigator (DP) providing feedback.

During the first two weeks, the principal investigator observed and supported the teachers to ensure protocol adherence through “assisted delivery”.^16^ Teachers, students, and parents could withdraw from the study at any time without penalty. No additional risks beyond everyday life were anticipated.

### 2.4 Control group

CG classes continued with their usual teaching and learning. They did not receive the IcPAB within the intervention period. After the post-tests, CG teachers received the resources to implement IcPAB. During the intervention, CG's teachers were contacted once weekly via WhatsApp and twice a week in person to obtain information on their lesson delivery patterns, to ensure that the CG did not receive any interventions.

### 2.5 Data collection

Data collection occurred at baseline in mid-July 2022 and at the end of the 12-week intervention in late-October 2022. Trained assistants who were blinded to IG allocation at baseline collected data. Prior to this study, a pilot study was conducted in a different school setting to assess the feasibility and administrability of the data collection procedures within the Sri Lankan Fifth-grade classroom context. The pilot implementation confirmed the practicality of administering the academic, anthropometric, fitness, stress, and movement behaviour assessments and supported the feasibility of collecting data within the intended school environment.

**Academic achievement:** This was the primary outcome of the intervention. Based on the interviews conducted with 21 teachers from the nine provinces of Sri Lanka (interview findings were published elsewhere)^18^ Mathematics and reading were identified as the most important subjects for students to perform well in the Grade Five national-level scholarship examination. Not only in the Sri Lankan context but also in the international context, it is evident that mathematics and reading performance are the key pillars of elementary education's academic achievement ^12^^,^^26^, ^27^, ^28^, ^29^. Therefore, mathematics and reading performance were used as measures of academic achievement in this study. The mathematics and reading evaluations were conducted at a time specified by the classroom teachers at each measurement point (baseline and post-test).

Mathematics achievement was evaluated through a curriculum-based standardized test designed by the teaching officers experienced in Grade Five mathematics performance-related evaluation. This test consisted of 60 questions to evaluate the expected performance of a given term. The students had to complete the test within 45 min. The principal investigator, three research assistants, and the classroom teachers collaboratively administered the test. The assessment was administered under standard classroom examination conditions. Test papers were scored using a predetermined marking scheme, and total scores were calculated based on the number of correct responses, with higher scores indicating better mathematic performance.

Reading achievement was evaluated through a standardized reading test, specific to the Sri Lankan Grade Five curriculum. Three grade five teachers who were not exposed to the study chose two paragraphs to be used at baseline and at the end of the intervention to evaluate students' reading achievement. The paragraphs were around 200 to 250 words, and each student had to read them for 2 min. Reading performance was evaluated by a teacher who was not the student's classroom teacher (yet a classroom teacher at the same school's parallel grade) under the principal investigator's distant observation. The reading performance was evaluated based on four criteria such as pronunciation, reading speed, responding to punctuation marks, and loudness. Higher scores indicated better reading performance.

Movement behaviors such as light PA, MVPA, steps count, and sedentary school time including the health outcomes such as BMI, aerobic fitness, and perceived stress were evaluated as secondary outcomes.

**Movement behaviors:**

Objective data for the movements were measured during normal school hours using waist-worn accelerometers (GT3-X+ and wGt3X-BT triaxial model, ActiGraph LLC, Pensacola, Fla, USA). Accelerometer data were collected for a week at baseline and at the intervention period's post-test (week 13).

The teachers randomly distributed the accelerometers to the students on the first school day and collected them on the fifth school day of the week. The research team demonstrated to teachers and students how to wear accelerometers on the first day. However, due to the limited number of accelerometers, this study used a randomly selected subsample (n = 47) to collect data on all movement behaviours, following a previous research practice. 30, 31, 32 After accelerometers were randomly distributed, the teachers prepared a list assigning each student to a specific numbered accelerometer to ensure that each child wore the same device every day throughout all data collection stages. Then, the research team received another version of the list in which the students’ anonymous IDs were linked to the accelerometer. Absent students who were assigned to an accelerometer were also recorded by the classroom teacher, and a research assistant verified that data during the school visits. Based on previous research practices,^16^^,^^33^ accelerometer data, which were identified for wearing more than five school hours on at least one school day, were included in the analysis for intervention effects.^16^^,^^33^ Following common practices in studies involving children, non-wear time was defined as 20 consecutive minutes of zeros.^16^^,^^34^^,^^35^ Freedson cut points were used to classify movement behavior intensities based on the data collected in 15-s epochs.^16^^,^^36^ Due to the focus on in-school physical activity, a longer wear time was not deemed necessary for the current study, as recommended by recent scholars.^37^ To confirm in-school wear-time for valid days, a further visual check of each accelerometer profile was undertaken.^37^ Therefore, research assistants randomly visited the schools during the data collection week to ensure that students wore the devices accurately during school hours.

**Health Outcomes**: BMI changes from baseline to the post-test were measured using anthropometry. Students' weight in kilograms to the nearest 0.1 kg 37, 38, 39 and height in centimeters to the nearest 0.1 cm^37^^,^^38^^,^^40^ was recorded by using a standard stadiometer and a weighing scale. Body weight in kilograms was divided by height in meters squared to measure the BMI.^37^^,^^38^^,^^40^ Students’ age- and gender-specific BMI categories were then identified using the calculator developed by the Ministry of Health in Sri Lanka.^41^

The multistage shuttle run test/beep test^42^ was used to measure aerobic fitness.^26^^,^^42^ Results from the test were used to calculate VO_2_ max using the equation proposed by previous studies.^43^^,^^44^ Students were asked to run back and forth on a 20 m course in response to a sound signal emitted from a pre-recorded tape, ensuring they touched the 20 m line with their foot.^26^ The sound signal frequency increases by 0.5 km/h per minute, indicating the next stage (level), starting at 8.5 km/h. The assessors verbally encouraged the participants to reach the end lines when they were running. The test ended when participants were unable to maintain the required pace within the given time.

Perceived stress was measured using an adapted and translated Sinhala version of the Perceived Stress Questionnaire 8–11 (PSQ8-11), a self-reported instrument specifically designed to measure psychological and physiological stress among children aged 8–11 years. The Sinhala version utilised in the present study was previously translated, culturally adapted, and psychometrically validated among Sri Lankan primary school children and the procedures were published elsewhere.^43^ PSQ8-11 measured two subscales: perceived psychological stress and physiological stress. Each subscale consisted of 10 questions requiring the students to recall their feelings from the previous week. Students self-rated their responses to 20 questions on a four-point Likert scale (1 = never, 2 = sometimes, 3 = often, 4 = very often). A higher score on the questionnaire indicates greater perceived stress.^43^ To the best of this principal investigator's knowledge, there was little to no questionnaire tool to evaluate the perceived stress of children under 11 years old in Sri Lanka. Thus, the principal investigator adapted and translated PSQ8-11 and analyzed its psychometric properties. The findings of the psychometric properties of the translated Sinhala version of were presented elsewhere.^45^

### 2.6 Process evaluation as an indicator of the feasibility of the IcPAB

The feasibility of the IcPAB in this study was based on a formative process evaluation,^46^^,^^47^ which includes intervention dose, intervention fidelity, and participants' perceptions of engagement and acceptability throughout the 12-week intervention period.

Intervention dose: Intervention dose referred to the quantity of the intervention delivered during the study period, specifically the frequency of IcPAB implementation within classrooms. To assess dose, class teachers from the three Grade Five intervention classrooms maintained weekly implementation records documenting the number of IcPAB sessions conducted. These records enabled the research team to quantify the extent to which the intervention was delivered across the 12-week period.

Intervention fidelity: Intervention fidelity referred to the extent to which the IcPAB programme was implemented according to the intended intervention procedures. Fidelity monitoring focused on the use of prescribed IcPAB activity cards and the consistency of intervention delivery across classrooms. Teachers recorded how frequently each IcPAB card was used during each intervention week, enabling evaluation of adherence to the planned classroom-based physical activity structure and academic integration components.

The intervention was implemented among three Grade Five classrooms comprising approximately 30–35 students per class. However, individual student attendance and participation exposure were not monitored throughout the intervention period. Therefore, the dose and fidelity findings primarily reflect classroom-level implementation patterns rather than individual-level participation rates.

Teachers' logbooks were the primary tool for assessing fidelity, with teachers recording the number of IcPAB sessions delivered daily. They were reminded via daily WhatsApp messages, and a research team member visited the schools weekly to ensure logbooks were properly filled.

Feasibility and acceptability: Random feedback was collected throughout the intervention so that the teachers could share feelings of their experience on using the IcPAB cards, class management, and student engagement. At the same time, students gave verbal feedback weekly on the enjoyment and engagement of the activities using two closed-ended questions: 1) Did you enjoy the activities today? The answer is Yes or No; 2) Was it easy to follow the instructions and do the activities today? The answer is Yes or No. This feedback was collected primarily to support ongoing intervention implementation, maintain participant engagement, and identify practical classroom-related challenges during the 12-week intervention period. The intention of collecting such feedback was to pragmatically provide implementation support and make minor practical adjustments where necessary. Overall, students generally reported positive engagement with the activities throughout the intervention period. However, teachers occasionally reported practical classroom-space limitations during certain movement activities involving larger body movements. In such situations, teachers sometimes asked a small number of students to temporarily move into open spaces between desk rows to comfortably perform the activities. After the intervention, a 5-point Likert-scale questionnaire was distributed to teachers and students for process evaluation (Supplementary file 2). The teachers' questionnaire had 8 items, and the students’ had 9 items, both adapted from previous research.^19^^,^^48^ These questionnaire data were used to evaluate the process quantitatively.

### 2.7 Data analysis

Data were analyzed using IBM SPSS software version 27. Given the school-level randomization, primary analyses were conducted at the individual level, with results interpreted as exploratory. Where appropriate, mixed-effects models with random intercepts were used to account for repeated measures within individuals. Due to the study's preliminary nature and the multiple outcomes assessed, no formal multiplicity adjustment was applied. Findings are therefore presented as hypothesis-generating rather than confirmatory.

A randomization check was performed using independent t-tests for continuous outcomes and chi-square tests for categorical variables. If significant differences were found between the IG and CG at baseline, these variables were included as covariates in subsequent analyses to account for potential confounding effects, ensuring a more accurate assessment of intervention outcomes. Since the schools were from the same geographical area and had similar socioeconomic statuses, baseline differences were expected to be minimal. Descriptive statistics, including mean (M), standard deviation (SD), and percentages, were used to summarize the baseline characteristics of the sample.

The primary analysis used a modified intention-to-treat (m-ITT) approach.^49^ There was no missing data from dropouts, and the missing data were classified as completely random (MCAR) with an insignificant test result (X^2^_Little's MCAR_ = 266.039, df = 702, *P* = 1.000). Missing data ranged from 2.7% to 8.2% and were handled using multiple imputations with series mean.

To assess the intervention's effectiveness, generalized linear mixed models (GLMM) were used, linking dependent variables at both the individual (student) and group (intervention vs. control) levels with time (pre- and post-test).^16^^,^^50^^,^^51^ A random intercept was included to account for repeated measures.^52^ Moderation effects by gender were tested using an interaction term in the GLMM (group × time × gender).^52^ Statistical significance was set at a 5% level (two-tailed).^53^ Intervention effects were assessed using type III fixed-effect tests from the GLMM. Effect sizes were interpreted based on Cohen's *d* and β values and the 95% confidence intervals were reported.^30^^,^^38^ A sensitivity analysis was conducted to check the robustness of results, using only participants who completed both baseline and post-test measurements (i.e., per-protocol analysis). Movement behavior-related data from 92 students who had completed all the baseline and post-test measurements were included in the analysis.

## 3 Results

**Fig. 2:**
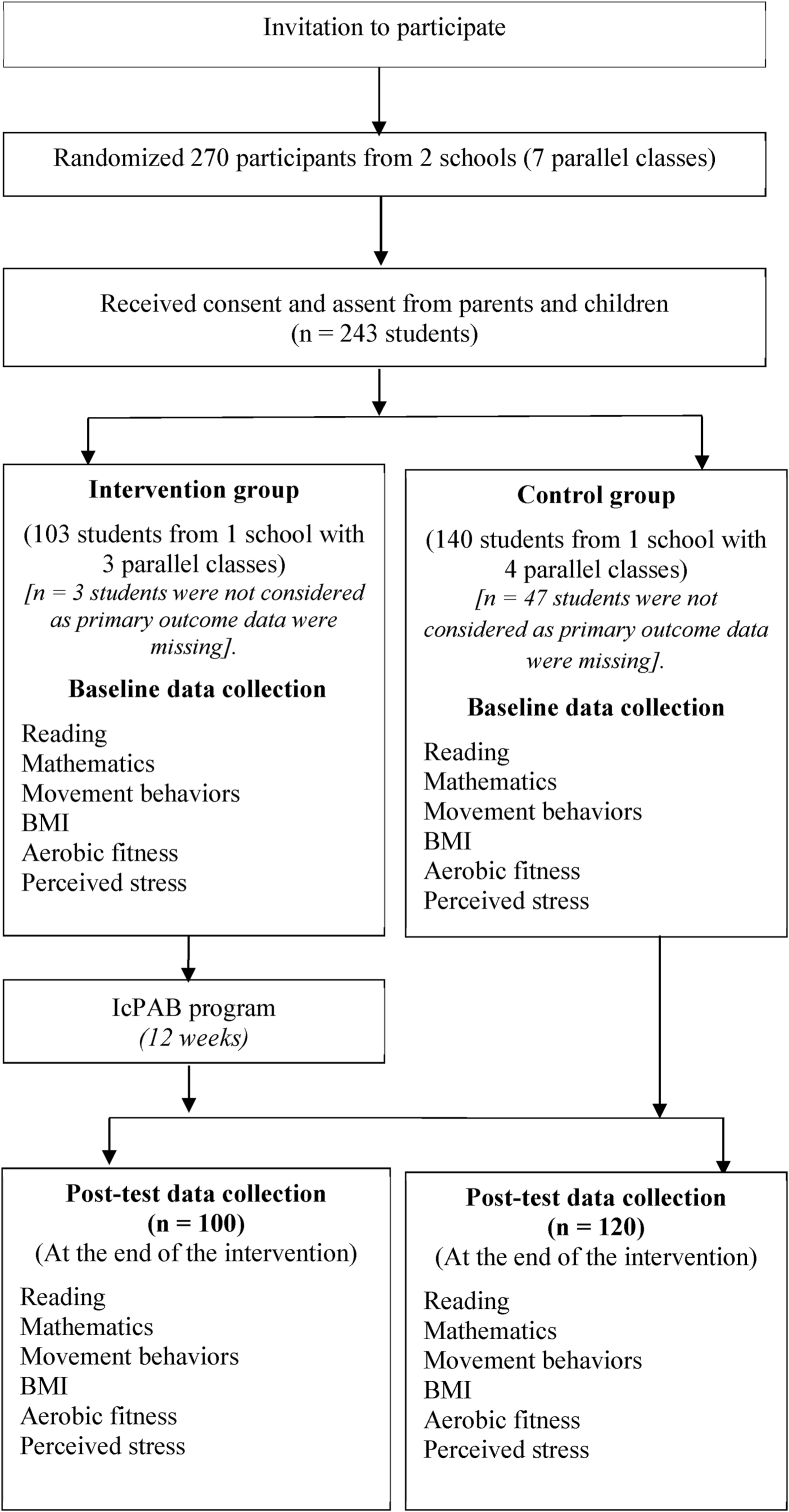
Study flow diagram.

Participants' flow through the study for all outcomes is reported [Fig. 2]. During the intervention adverse events were not reported. The sample's age ranged from 9 to 10 years old (Mean = 9.5 years ± 0.5). More than half of the participants were the boys (59.5%) (Table 1). The IG had 103 fifth-grade students across 3 classrooms. The CG had 167 students across 4 parallel classrooms. Baseline data from three IG students and 47 CG students could not be collected, and they were not included in the analyses. Hence, 220 students (IG = 100, CG = 120) were included in the analysis. In the movement behavior-related data analysis, data from two students did not meet the wear-time requirements at baseline; thus, 92 students (IG = 46; CG = 46) were included. Baseline movement behaviour data indicated that the CG had higher physical activity levels compared to the IG. In the movement behaviour subsample, the CG demonstrated higher baseline MVPA (31.1 ± 8.4 min/week) and higher step counts (7998 ± 1023 steps/week) than the IG (27.2 ± 9.4 min/week; 5609 ± 1019 steps/week), indicating some baseline imbalance between groups despite classroom-level randomization.

**Table 1:** Baseline characteristics.

Characteristics	Total: M (SD) N = 220	IG: M (SD) N = 100	CG: M (SD) N = 120
Gender			
Boys (n [%])	131 (59.5%)	65 (65%)	66 (55%)
Girls (n [%])	89 (40.5%)	35 (35%)	54 (45%)
Age (range 9 to 10 years) (M ± SD)	9.4 (0.5)	9.5 (0.5)	9.4 (0.5)
**Academic Achievement**			
Reading (*score out of 100*)	79.2 (12.4)	82.2 (13.9)	76.7 (10.5)
Mathematics (*score out of 100*)	82.0 (14.3)	80.8 (16.3)	83.1 (12.3)
**Movement behavior^b^ (Sub-sample: n = 9**2)			
Light PA (min/week)	283.0 (36.4)	282.1 (47.9)	283.9 (19.4)
MVPA (min/week)*	29.2 (9.1)	27.2 (9.4)	31.1 (8.4)
Step counts (steps per week)*	6803 (1573.0)	5609 (1019.3)	7998 (1023.0)
Sedentary school time (min/week)	1487.8 (40.7)	1490.7 (53.1)	1485.0 (22.9)
**Health Outcomes**			
Body-mass-index (kg/m^2^)	15.7 (2.6)	16.0 (2.7)	15.5 (2.6)
Aerobic fitness (*ml/kg/min)*	19.8 (1.3)	20.0 (1.5)	19.6 (1)
Perceived stress *(total score out of 4)*	1.8 (0.4)	1.8 (0.4)	1.8 (0.4)
Perceived psychological stress	1.9(0.4)	1.9 (0.4)	1.9 (0.4)
Perceived physiological stress	1.7(0.5)	1.7 (0.5)	1.8 (0.5)

Note:*P < .05;^a^Sample of movement behaviours = 92 (IG = 46, CG = 46); IG = intervention group; CG = control group; SD = standard deviation; PA = physical activity; MVPA = moderate-to-vigorous physical activity; ^b^The subsample was consisted of 55 boys(59.8%) and 37 girls (40.2%) (IG – 28 and 18, CG – 23 and 19 boys and girls respectively).

### 3.1 Intervention effects

**Fig. 3:**
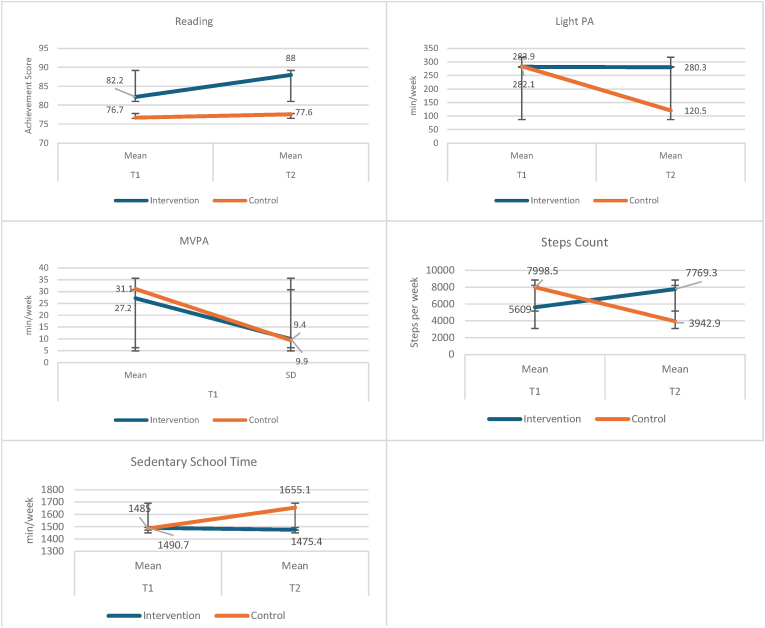
Means of the variables with significant intervention effects at T1 and T2.

Fig. 3 and Table 2 shows the intervention effects (time×group) and their moderation by gender (time×group×gender) on all outcomes relative to the control group. Figure panel 3 shows the mean changes of the variables which showed statistically significant intervention effects.

**Table 2:** Results of the intervention effects and the moderation effects by gender (N = 220).

Outcome	Mean (SD) at T1		Mean (SD) at T2		Time×group^1^		β [95% CI]	Time^1^		Group^1^		Time×group×Gender^1^	
	IG	CG	IG	CG	*F/χ*^2^ test (*df*)	*P* value		*F/χ*^2^ test (*df*)	*P* value	*F/χ*^2^ test (*df*)	*P* value	*F/χ*^2^ test (*df*)	*P* value
Reading^a^ (Cohen's *d* = 0.5)	82.2 (13.9)	76.7 (10.5)	88.0 (12.9)	77.6 (13.0)	15.8 (1208)	**<.001**	4.6 [1.5,7.7]	33.0 (1208)	<0.001	32.5 (1208)	<0.001	0.1(1208)	0.752
Mathematics (Cohen's *d* = 0.1)	80.8 (16.3)	83.1 (12.3)	68.9 (17.2)	69.4 (19.1)	0.7 (1216)	0.384	3.5 [-1.3,8.3]	170.3 (1216)	<0.001	0.2 (1216)	0.657	0.9 (1216)	0.346
Light PA (Cohen's *d* = 1.9)	282.1 (47.9)	283.9 (19.4)	280.3 (53.9)	120.5 (36.3)	185.0 (1,88)	**<.001**	141.4 [110.4172.3]	170.4 (1,88)	<0.001	160.3 (1,88)	<0.001	4.4 (1,88)	**0.039**^b^
MVPA^a^ (Cohen's *d* = 1.2)	27.2 (9.4)	31.1 (8.4)	44.3 (9.9)	24.3 (9.4)	76.2 (1,85)	**<.001**	22.4[15.1,29.7]	12.9 (1,85)	<0.001	36.9 (1,85)	<0.001	0.7 (1,85)	0.356
Step count^a^ (Cohen's *d* = 2.571	5609.0 (1019.3)	7998.5 (1023.0)	7769.3 (1339.0)	3942.9 (1046.3)	357.2 (1,85)	**<.001**	6051.1 [5186.9,6915.2]	32.1 (1,85)	<0.001	16.7 (1,85)	<0.001	1.4 (1,85)	0.244
Sedentary school time (Cohen's *d* = 1.9)	1490.7 (53.1)	1485.0 (22.9)	1475.4 (59.4)	1655.1 (40.8)	202.1 (1,88)	**<.001**	−162.7 [-196.7, −128.8]	123.1 (1,88)	<0.001	155.0 (1,88)	<0.001	4.6 (1,88)	**0.035**^c^
BMI (Cohen's *d* = 0.1)	16.0 (2.7)	15.5 (2.6)	15.1 (2.5)	15.0 (3.0)	0.8 (1216)	0.380	−0.2 [-0.8,0.5]	23.0(1216)	<0.001	0.7(1216)	0.399	0.0 (1216)	0.848
Aerobic fitness (Cohen's *d* = 0.2)	20.0 (1.5)	19.6 (1)	20.5 (1.4)	19.8 (1.2)	2.9 (1216)	0.088	0.2 [-0.4,0.7]	12.3 (1216)	<0.001	9.9 (1216)	0.002	0.8 (1216)	0.365
Perceived stress (Cohen's *d* = 0.1)	1.8 (0.4)	1.8 (0.4)	1.6 (0.4)	1.6 (0.4)	0.5 (1216)	0.499	0.0 [-0.1,0.2]	58.1 (1216)	<0.001	0.8(1216)	0.364	0.0 (1216)	0.916
Perceived psychological stress (Cohen's *d* = 0.1)	1.9 (0.4)	1.9 (0.4)	1.7 (0.5)	1.7 (0.4)	0.7 (1216)	0.416	0.0 [-0.1,0.2]	32.6 (1216)	<0.001	0.0(1216)	0.925	0.0 (1216)	0.869
Perceived physiological stress (Cohen's *d* = 0.0)	1.7 (0.5)	1.8 (0.5)	1.5 (0.4)	1.6 (0.4)	0.1 (1216)	0.809	0.0 [-0.1,0.2]	41.61 (1216)	<0.001	2.7(1216)	0.100	0.1 (1216)	0.748

Note. ^1^Type III tests; LPA = light physical activity (min/week); MVPA = moderate-to-vigorous physical activity (min/week); BMI = body-mass-index; SD = standard deviation; df = degree of freedom; IG = intervention group; CG = waitlist control group; T2 = post-intervention assessment; ^a^ = adjusted for baseline differences; D = Type III fixes effect test statistic; β = estimated intervention effect at T2; CI = confidence interval; IG, T2 and male students are the reference categories; ^b^:(β = 51.6, 95% CI [2.8100.4]); ^c^:(β = −57.8, 95% CI [-111.4,-4.2).

*Academic achievement:* A significant group × time interaction was observed for reading (F(1,208) = 15.8, p < .001), indicating that reading scores increased in the intervention group from baseline (M = 82.2, SD = 13.9) to post-intervention (M = 88.0, SD = 12.9), whereas scores remained relatively stable in the control group (baseline M = 76.7, SD = 10.5; post M = 77.6, SD = 13.0). The estimated intervention effect at post-test was β = 4.6 [1.5, 7.7], confirming a positive intervention effect on reading performance. In contrast, no significant group × time interaction was observed for mathematics (F(1,216) = 0.7, p = .384), indicating no differential score change between groups over time, although descriptive means suggest a decrease in both groups over time from T1 to T2.

*Movement behaviors:* Significant group × time interactions were observed for all indicators, including light PA, MVPA, step counts, and sedentary school time (all p < .001). These results indicate clear divergence between groups over time, with the intervention group showing improvements in physical activity levels and reductions in sedentary time, while the control group showed either smaller changes or opposite trends. For example, MVPA increased in the intervention group (27.2 ± 9.4 to 44.3 ± 9.9 min/week) but decreased in the control group (31.1 ± 8.4 to 24.3 ± 9.4 min/week), with a significant interaction effect (F(1,85) = 76.2, p < .001; β = 22.4 [15.1, 29.7]). A statistically significant group × time × gender interaction was found for light PA (F(1,88) = 4.4, p = .039). For sedentary school time statistically significant group × time × gender interaction was found (F (1,88) = 4.6, *p* = .035). und (found (These three-way interactions indicated a significant reduction in sedentary time at school among IG boys compared with IG girls.

*Health outcomes:* No significant time × group interaction for BMI (F(1,216) = 0.8, *p* > .05), aerobic fitness (F (1,216) = 2.9, *p* > .05) and perceived stress (F(1,216) = 0.5, *p* > .05) outcomes were found.

Intervention effects were not modified by students’ gender, except for LPA and sedentary school time. The sensitivity analyses confirmed the main analyses' findings (Supplementary File 3).

### 3.2 Process evaluation

#### 3.2.1 Intervention dose and fidelity

The minimum weekly implementation of the IcPAB was 7 times, and the maximum was 23 times. As a result, the daily dosage of IcPAB ranged from 1.4 times (7/5) to 4.6 times (23/5). The number of implemented IcPAB activities varied by class. I.e., the Teacher of class One did two to five IcPAB daily, while the other teachers implemented two to three activities. Hence, the teachers used the cards at least two times daily (Supplementary file 4). In addition, teachers did not use all the IcPAB cards during the intervention. The average number of cards selected was five to 15 out of 20.

#### 3.2.2 Evaluation on engagement and acceptability with IcPAB

90 IG students (58 boys (64.4%), 32 girls (35.6%)) and three teachers (2 males and 1 female) responded to the process evaluation questions (Table 3). The results highlight the respondents' positive engagement and acceptability with the IcPAB. With reference to ‘very strongly agreeing’, 98.9% happily participated, and 92.2% expressed interest in future participation. Further, most felt happier (86.7%) and understood instructions easily (88.9%). 73.3% found IcPAB manageable. 70% felt they had enough space for IcPAB, and fewer (46.7%) thought the allocated time was sufficient. Only 45.6% felt they engaged in enough activity daily.

**Table 3:** Feedback.

Evaluation on engagement and acceptability with IcPAB (students)	Very strongly agreed	Agreed	Neutral	Did not agree	Did not agree at all
I happily engaged in the IcPAB activities	89 (98.9%)	1 (1.1%)	-	-	-
I like to IcPAB in future	83 (92.2%)	5 (5.6%)	2 (2.2%)	-	-
I felt happier than before	78 (86.7%)	11 (12.2%)	1 (1.1%)	-	-
I could easily do IcPAB	66 (73.3%)	19 (21.1%)	1 (1.1%)	1 (1.1%)	3 (3.3%)
I easily understood instructions	80 (88.9%)	6 (6.7%)	3 (3.3%)	1 (1.1%)	-
I had enough space for IcPAB	63 (70%)	14 (15.6%)	2 (2.2%)	3 (3.3%)	8 (8.9%)
Allocated time was enough	42 (46.7%)	6 (6.7%)	5 (5.6%)	-	37 (41.1%)
I did enough IcPAB everyday	41 (45.6%)	6 (6.7%)	5 (5.6%)	4 (4.4)	34 (37.8)
The speed of IcPAB was enough	72 (80%)	3 (3.3%)	5 (5.6%)	2 (2.2%)	8 (8.9%)

**Evaluation on engagement and acceptability with IcPAB (teachers)**	Very strongly agreed	Agreed	Neutral	Did not agree	Did not agree at all

IcPAB fitted well to the routine	3 (100%)	-	-	-	-
IcPAB were easy to adapt	2 (66.7%)	1 (33.3%)	-	-	-
IcPAB created many additional work	-	-	2 (66.7%)	-	1 (33.3%)
IcPAB was not flexible	-	-	1 (33.3%)	1 (33.3%)	1 (33.3%)
IcPAB had much to do	-	-	2 (66.7%)	-	1 (33.3%)
Had to allocate too long time	-	-	-	-	3 (100%)
I will use IcPAB in future	3 (100%)	-	-	-	-
I recommend IcPAB to others	3 (100%)	-	-	-	-

All teachers agreed that IcPAB fit well into the daily classroom routine and were easy to adapt. Two teachers were neutral about additional workload and program demands, while one felt this was not a problem. Two teachers found IcPAB flexible. All teachers expressed willingness to use IcPAB in the future and recommended it to colleagues.

## 4 Discussion

This study evaluated the preliminary effects and feasibility of a 12-week, teacher-led, 5-min IcPAB on academic achievement, movement behaviors, and health outcomes among Sri Lankan fifth graders. The findings indicate that IcPAB is a feasible school-based intervention that can be implemented in examination-oriented classroom environments while producing meaningful improvements in selected academic and physical activity outcomes, without adversely affecting health indicators. Academic outcomes: A key finding of this study was the significant improvement in reading achievement in the intervention group compared to the control group. This aligns with previous randomized controlled trials^54^ and a meta-analytic evidence,^18^ suggesting that classroom-based physical activity interventions can positively influence reading-related cognitive outcomes. The observed improvement may be because the teachers might have used more reading-related cards. Reading skills might be more responsive to the IcPAB within a shorter period based on the cognitive and behavioral impacts such as improved on-task behavior.^35^^,^^55^ In contrast, significant group-by-time interaction was not observed for mathematics achievement. This finding aligns with previous studies^29^^,^^35^ and a meta-analysis.^18^ One possible explanation is increased post-test difficulty, as mean scores were lower at post-intervention in both groups, suggesting higher task complexity rather than intervention effects.

Movement behaviours: The intervention produced strong and consistent effects on movement behaviors with large effect sizes observed for light PA, MVPA, step counts, and sedentary school time. Especially, the findings on MVPA align with previous reviews.^18^^,^^56^^,^^57^ The IG increased their MVPA from 27.2 to 44.3 min while the CG declined their MVPA from 31.1 to 24.3 min close to the time of the national examination. The net difference is around 20 min in favor of the IG. When expressed in absolute terms, these changes corresponded to several additional minutes of MVPA per school day. Contributing new knowledge, this divergence is particularly noteworthy given that post-intervention data collection coincided with preparation for the Grade 5 national examination, a period typically associated with increased sedentary classroom instruction.^13^^,^^20^ In this context, IcPAB appears to have played a protective role in maintaining physical activity levels despite heightened academic demands. Also, this study adds to the limited evidence suggesting that IcPABs may influence light physical activity during school hours, specifically among boys. This gender specific difference may be due to the differences in how boys and girls respond to IcPAB or the cultural perceptions of engaging in PA.^58^ These exploratory findings indicate the need for future research to examine gender-related differences in intervention response,^58^^,^^59^ potentially using behavioral frameworks such as COM-B to inform inclusive intervention design.

Heath-related outcomes: Intervention effects were not observed for BMI. The absence of significant BMI changes was not unexpected, considering the relatively short duration and low-volume nature of the IcPAB intervention. Similar findings have been reported by Drummy and colleagues in 2016 who observed no significant effects on adiposity outcomes following a 12-week classroom activity break intervention, which quoted in a previous review.^18^ The results were also likely influenced by Sri Lanka's economic crisis and the COVID-19 pandemic, 60, 61, 62 during which malnutrition among school students was reported by UNICEF,^63^ and other sources. 64, 65, 66 However, BMI is a crude measure of body weight relative to height and does not distinguish between muscle mass and fat mass. Hence, IcPAB might not adversely affect BMI, given the short duration of the intervention and the multifactorial nature of weight-related outcomes. The intervention may increase muscle mass without significantly affecting BMI. This highlights the need for further studies that consider nutrition and socio-economic factors, especially in low-and-middle income countries where malnutrition may be present. Because IcPAB alone may not be sufficient to significantly impact BMI or muscle mass, factors such as dietary habits, genetics, and individual variation also play a role.^67^^,^^68^ Future studies incorporating direct measures of body composition, such as bio-electrical impedance analysis, could provide more detailed insights.

Aerobic fitness indicated a non-significant limited effect of IcPAB on cardiovascular fitness. This could be due to a relationship between the students’ growth factors and their fitness levels.^69^ For example, previous research has shown that as kids age, their fitness levels naturally increase.^70^ Yet, more studies with longer follow-up periods are needed for a robust conclusion.

Perceived stress and its sub-were not significantly affected by the intervention. A meta-analysis, which was related to exam-related anxiety, showed similar findings.^18^ Also, perceived stress was relatively low in the IG [ (Mean 1.8 ± 0.4) at the pre-test. This indicates that the sample had limited room for improvement in perceived stress. Alternatively, the duration and frequency of the IcPAB may have been insufficient to meaningfully affect the perceived stress. Or the reliance on student perception data may have caused a reporting bias. Therefore, further exploration of these relationships is warranted in future interventions.

Feasibility: A key contribution of this study is demonstrating the feasibility of teacher-led IcPAB implementation in a highly examination-driven educational environment. Teacher logs indicated an average of 2.7 IcPAB sessions per day. Fidelity data indicated a closer adherence to the intended activity implementation requirement. Previous findings,^18^^,^^19^^,^^28^ revealed that teachers faced challenges in consistently implementing IcPAB 3 times daily. Process evaluations findings further support the acceptability of the intervention. A higher engagement and greater acceptability of IcPAB was noted, with teachers' preferences for ≤5 min shorter IcPAB despite strong curricular demands associated with preparation for the Grade 5 scholarship examination. Hence, the findings support the continued use of the COM-B framework for IcPAB adaptation and teacher support, specifically in low- and middle-income country settings.

Although the process evaluation findings indicated generally acceptable implementation fidelity, several practical considerations emerged that may inform future intervention refinement. Teachers reported that maintaining regular IcPAB implementation became more challenging during periods of intensive examination preparation and competing academic demands. Certain activity cards appeared to be preferred over others, potentially reflecting differences in classroom feasibility, ease of delivery, or student engagement. Occasional classroom management difficulties and limited physical classroom space were also identified as minor barriers to implementation. These findings suggest that future IcPAB programmes may benefit from greater flexibility in activity selection, additional teacher training, and adaptation to classroom environmental constraints within examination-oriented school settings.

However, this study has several limitations. Data were collected only from Sinhala-speaking fifth graders and teachers at two government schools in Uva Province. This limited the findings’ generalizability to other Sri Lankan ethnic groups or other low-and-middle-income countries. The severity of the COVID-19 pandemic^60^^,^^61^ in other provinces, constrained the participant selection.^13^ Intervention school teachers indicated that detailed time recording could increase workload and reduce feasibility in classroom settings. Hence, the duration of each IcPAB session was not systematically recorded, as the monitoring system was intentionally simplified to reduce teacher burden and improve adherence. This is acknowledged as a limitation, and future studies should consider incorporating more detailed fidelity measures, including session duration. Although the intervention group included a greater proportion of boys than girls, the present study was not designed to examine factors influencing programme recruitment, participation, or engagement by gender. Furthermore, the process evaluation instruments did not specifically assess gender-related barriers or facilitators to participation. Therefore, it was not possible to determine whether the observed gender imbalance reflects differences in activity preferences, perceptions of classroom-based physical activity, sociocultural influences, or other contextual factors. Future studies should explicitly examine reach-related outcomes and investigate gender-specific factors influencing participation and engagement to support equitable implementation of IcPAB programmes.

Importantly, given the between-school cluster design, potential confounding due to unmeasured school-level differences such as teacher effectiveness, classroom practices, and institutional environment cannot be fully excluded. Although baseline characteristics between the intervention and control schools were broadly comparable for most key variables, and statistical analyses adjusted for baseline values using mixed-effects models, residual confounding at the school level may still have influenced the observed outcomes. The small number of participating schools further limited cluster-level inference and statistical power.

This study examined the immediate effects but not the residual effects through follow-ups. Interesting additional factors influencing academic performance, such as cognitive ability,^18^^,^^71^^,^^72^ dietary practices,^73^ and sleeping^74^ were not assessed in this study due to time and budget constraints. Additionally, the small number of participating schools limited cluster-level inference and statistical power. These limitations underscore the preliminary nature of the study and highlight the need for larger, multi-school clustered trials with follow-up period and standardized outcome measures.

A further limitation of the present study was that accelerometer-based movement behaviour assessment was limited to school hours. This decision aligned with the primary scope of the study, which focused specifically on the within-school impact of IcPAB implementation in examination-oriented classroom settings where prolonged sedentary behaviour was identified as a key concern. However, future studies may benefit from assessing whole-day movement behaviours to determine whether participation in classroom-based physical activity interventions influences physical activity patterns beyond the school environment. Also, the movement behaviour assessment was limited to a subsample of participants because the number of available accelerometers was insufficient to monitor all students. Although the subsample included equal numbers of students from the intervention and control groups, the restricted device availability reduced the representativeness of the objectively measured movement behaviour data and limited statistical power for these outcomes. Future studies should seek additional funding and resources to facilitate accelerometer-based monitoring in larger samples and across a greater number of schools.

Despite its limitations, the present study provides practical insights into implementing IcPABs in academically intensive primary school settings. The findings suggest that short, teacher-led activity breaks can be integrated into daily classroom routines without compromising instructional time and may contribute to more active learning environments. Also, this study addressed several recommendations from previous research: (1) intervention materials were developed in collaboration with Sri Lankan fifth-grade teachers using the COM-B model, ensuring the program was both theory- and evidence-based,^18^^,^^29^ (2) gender-based moderation effects were analyzed,^18^^,^^29^^,^^57^ (3) provided evidence on the use of shorter, curriculum-integrated IcPAB that would be feasible,^16^^,^^29^^,^^75^ (4) fidelity and process evaluation mechanisms were followed,^16^^,^^29^ (5) curriculum-based measures were used,^16^^,^^29^ (6) objective measurements were used to analyze movement behaviors^16^^,^^76^ and (8) some evidence on the effects of academic load on stress 77, 78, 79 including under-researched areas ^43^^,^^77^^,^^80^, ^81^, ^82^, ^83^ such as body-mass-index and aerobic fitness were provided.

## 5 Conclusion

This study indicates that IcPAB is a feasible and acceptable strategy for increasing school-time movement behaviours among Sri Lankan fifth-grade students. Preliminary findings generate hypotheses that IcPAB interventions implemented within examination performance focused primary school settings may contribute to improvements in reading-related academic outcomes, school-time movement behaviours, cardiorespiratory fitness, and perceived stress among fifth graders. The findings also suggest the potential influence of contextual and gender-related factors on intervention responsiveness. Future large-scale, longer-term, and follow-up studies are therefore warranted to further examine the effectiveness, sustainability, and broader behavioural and psychosocial impacts of IcPAB interventions among primary school children.

## Authors’ contributions

DP (D.L.I.H.K. Peiris): Conceptualization, Data curation, Formal analysis, Funding acquisition, Investigation, Methodology, Project administration, Resources, Validation, Visualization, Writing – original draft, Writing – review & editing. YD (Yanping Duan): Conceptualization, Supervision, Writing – review & editing. CV (Corneel Vandelanotte): Resources, Supervision, Writing – review & editing. WL (Wei Liang): Methodology, Data curation, Formal analysis, Writing – review & editing.

## Competing interests

The authors declare that they have no competing interests.
