# Supplementary material for: A school-level randomized preliminary study of in-classroom physical activity breaks among fifth graders
Source: J Exerc Sci Fit. 2026 Jul 13;24(4):200498. doi: 10.1016/j.jesf.2026.200498 (PMC13400259; doi:10.1016/j.jesf.2026.200498)
Supplement: Multimedia component 1 [file mmc1.docx]

## Supplementary file 1: The completed CONSORT Checklist.


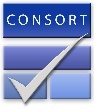
CONSORT 2010 checklist of information to include when reporting a randomised trial*

| Section/Topic | Item No | Checklist item | Reported on page No |
| --- | --- | --- | --- |
| Title and abstract | | | |
|  | 1a | Identification as a randomised trial in the title | 1 |
|  | 1b | Structured summary of trial design, methods, results, and conclusions (for specific guidance see CONSORT for abstracts) | 1 |
| Introduction | | | |
| Background and objectives | 2a | Scientific background and explanation of rationale | 1-4 |
|  | 2b | Specific objectives or hypotheses | 4 |
| Methods | | | |
| Trial design | 3a | Description of trial design (such as parallel, factorial) including allocation ratio | 4 |
|  | 3b | Important changes to methods after trial commencement (such as eligibility criteria), with reasons | N/A |
| Participants | 4a | Eligibility criteria for participants | 5 |
|  | 4b | Settings and locations where the data were collected | 4-5 |
| Interventions | 5 | The interventions for each group with sufficient details to allow replication, including how and when they were actually administered | 5-9 |
| Outcomes | 6a | Completely defined pre-specified primary and secondary outcome measures, including how and when they were assessed | 9-12 |
|  | 6b | Any changes to trial outcomes after the trial commenced, with reasons | N/A |
| Sample size | 7a | How sample size was determined | 4, 5 |
|  | 7b | When applicable, explanation of any interim analyses and stopping guidelines | N/A |
| Randomisation: |  |  |  |
| Sequence generation | 8a | Method used to generate the random allocation sequence | 4-5 |
|  | 8b | Type of randomisation; details of any restriction (such as blocking and block size) | 4-5 |
| Allocation concealment mechanism | 9 | Mechanism used to implement the random allocation sequence (such as sequentially numbered containers), describing any steps taken to conceal the sequence until interventions were assigned | 4-5 |
| Implementation | 10 | Who generated the random allocation sequence, who enrolled participants, and who assigned participants to interventions | 4-5 |
| Blinding | 11a | If done, who was blinded after assignment to interventions (for example, participants, care providers, those assessing outcomes) and how | 4-5 |
|  | 11b | If relevant, description of the similarity of interventions | N/A |
| Statistical methods | 12a | Statistical methods used to compare groups for primary and secondary outcomes | 12-13 |
|  | 12b | Methods for additional analyses, such as subgroup analyses and adjusted analyses | 13 |
| Results | | | |
| Participant flow (a diagram is strongly recommended) | 13a | For each group, the numbers of participants who were randomly assigned, received intended treatment, and were analysed for the primary outcome | 12-13 |
|  | 13b | For each group, losses and exclusions after randomisation, together with reasons | 8, 12 |
| Recruitment | 14a | Dates defining the periods of recruitment and follow-up | 6 |
|  | 14b | Why the trial ended or was stopped | 6 |
| Baseline data | 15 | A table showing baseline demographic and clinical characteristics for each group | 13-14 |
| Numbers analysed | 16 | For each group, number of participants (denominator) included in each analysis and whether the analysis was by original assigned groups | 13-14, 18 |
| Outcomes and estimation | 17a | For each primary and secondary outcome, results for each group, and the estimated effect size and its precision (such as 95% confidence interval) | 14, 17 Supplementary File 3. |
|  | 17b | For binary outcomes, presentation of both absolute and relative effect sizes is recommended | N/A |
| Ancillary analyses | 18 | Results of any other analyses performed, including subgroup analyses and adjusted analyses, distinguishing pre-specified from exploratory | 10,11 Supplementary File 3. |
| Harms | 19 | All important harms or unintended effects in each group (for specific guidance see CONSORT for harms) | 13 |
| Discussion | | | |
| Limitations | 20 | Trial limitations, addressing sources of potential bias, imprecision, and, if relevant, multiplicity of analyses | 21 |
| Generalisability | 21 | Generalisability (external validity, applicability) of the trial findings | 18-23 |
| Interpretation | 22 | Interpretation consistent with results, balancing benefits and harms, and considering other relevant evidence | 18-23 |
| Other information | | |  |
| Registration | 23 | Registration number and name of trial registry | 4 |
| Protocol | 24 | Where the full trial protocol can be accessed, if available | 5 |
| Funding | 25 | Sources of funding and other support (such as supply of drugs), role of funders | 23 |
|  |  |  |  |

Citation: Schulz KF, Altman DG, Moher D, for the CONSORT Group. CONSORT 2010 Statement: updated guidelines for reporting parallel group randomised trials. BMC Medicine. 2010;8:18.
© 2010 Schulz et al. This is an Open Access article distributed under the terms of the Creative Commons Attribution License (<http://creativecommons.org/licenses/by/2.0>), which permits unrestricted use, distribution, and reproduction in any medium, provided the original work is properly cited.

*We strongly recommend reading this statement in conjunction with the CONSORT 2010 Explanation and Elaboration for important clarifications on all the items. If relevant, we also recommend reading CONSORT extensions for cluster randomised trials, non-inferiority and equivalence trials, non-pharmacological treatments, herbal interventions, and pragmatic trials. Additional extensions are forthcoming: for those and for up-to-date references relevant to this checklist, see [www.consort-statement.org](http://www.consort-statement.org)

## Supplementary file 2: Process evaluation questionnaires.

**For Teachers: (English Version)**


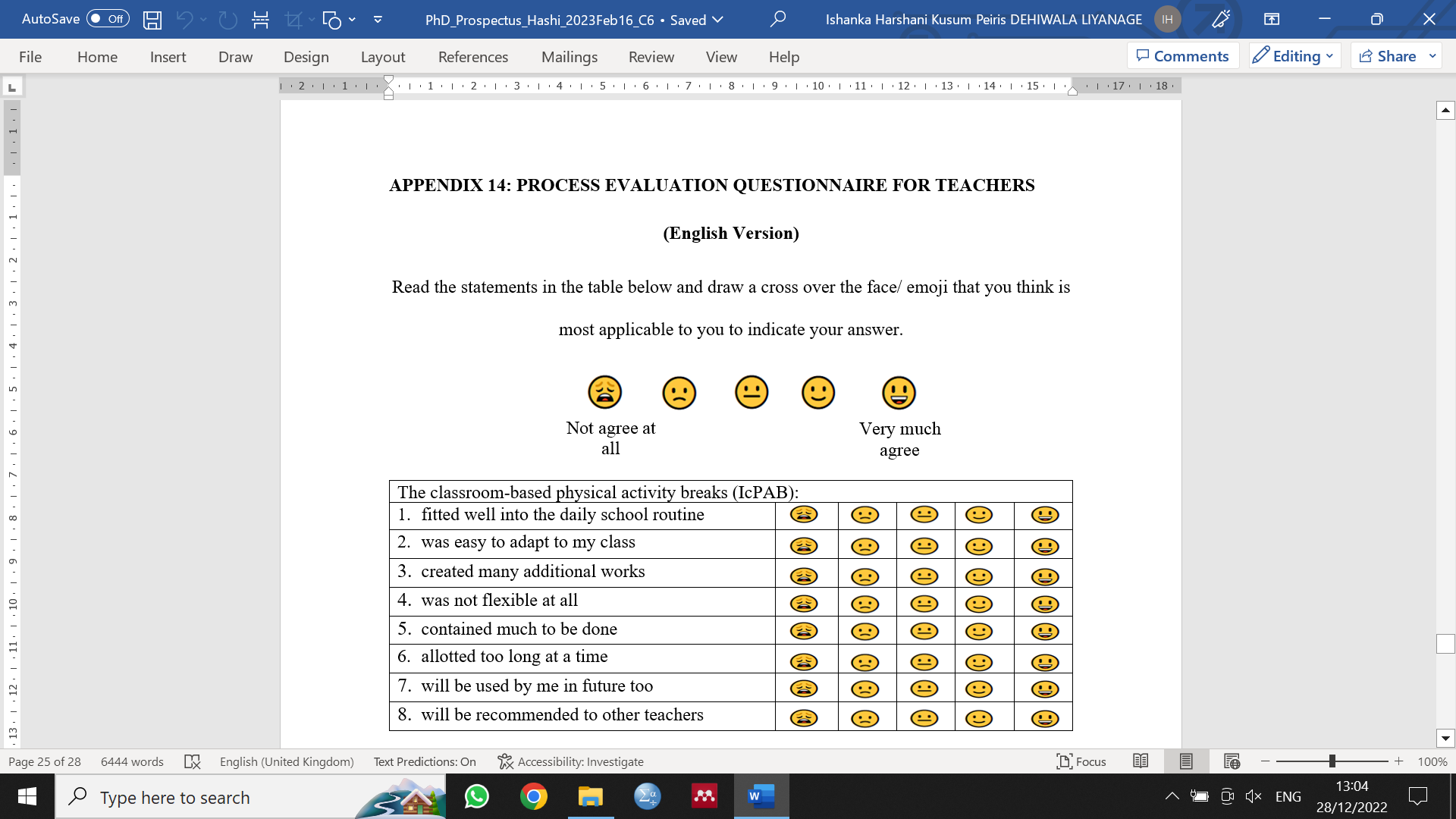


**For Students: (English Version)**


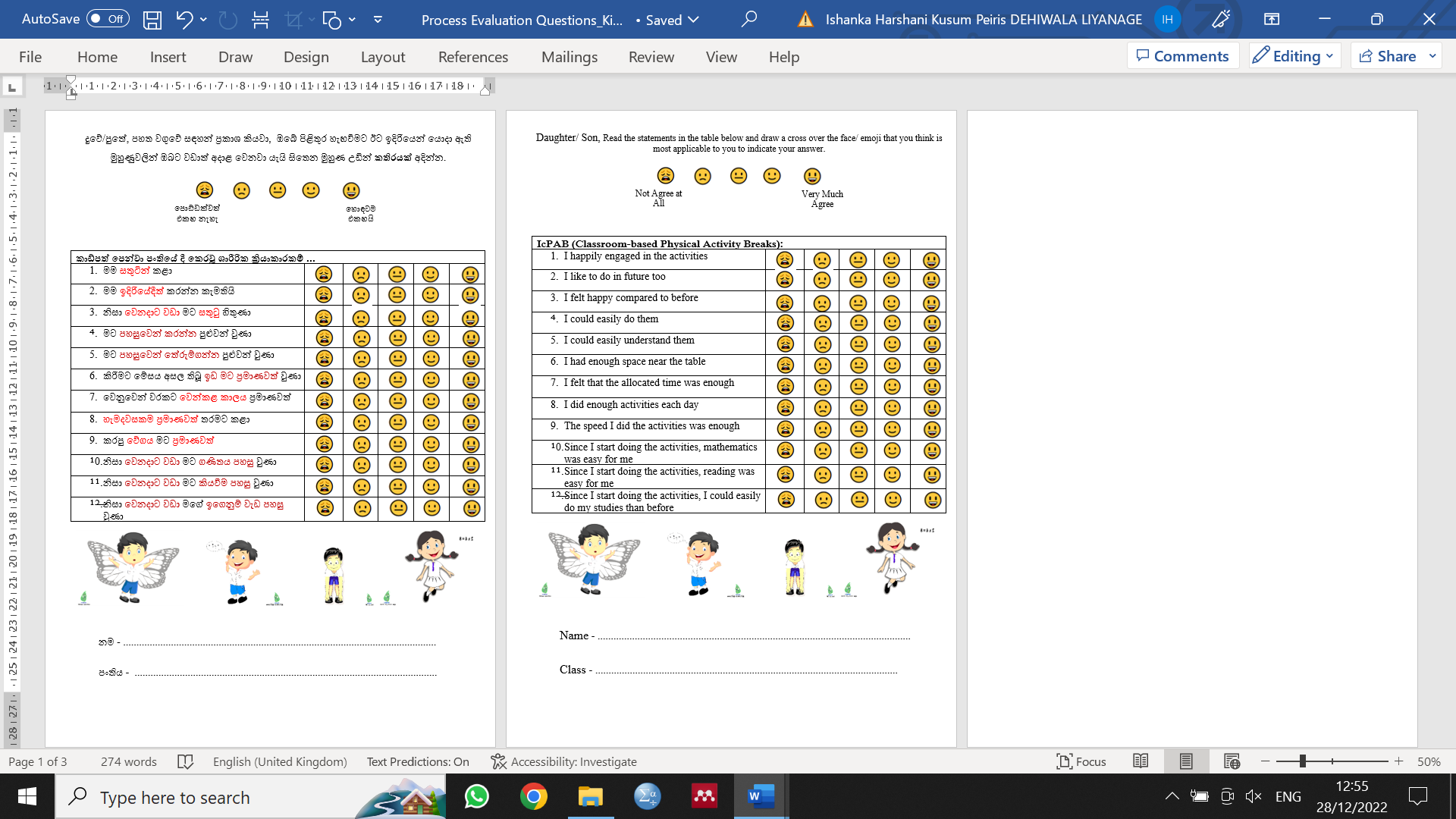


## Supplementary file 3: Baseline Characteristics

Table 1. Baseline characteristics

| **Characteristics** | **Total: M (SD)**  N = 220 | **IG: M (SD)**  N = 100 | **CG: M (SD)**  N = 100 |
| --- | --- | --- | --- |
| Gender  Boys (n [%])  Girls (n [%]) | 131 (59.5%)  89 (40.5%) | 65 (65%)  35 (35%) | 66 (55%)  54 (45%) |
| Age (range 9 to 10 years) (M+/- SD) | 9.4 (0.5) | 9.5 (0.5) | 9.4 (0.5) |
| **Academic Achievement** |  |  |  |
| Reading (*score out of 100*) | 79.2 (12.4) | 82.2 (13.9) | 76.7 (10.5) |
| Mathematics (*score out of 100*) | 82.0 (14.3) | 80.8 (16.3) | 83.1 (12.3) |
| **Movement behavior^b^ (Sub-sample: n = 9**2) |  |  |  |
| Light PA (min/week) | 283.0 (36.4) | 282.1 (47.9) | 283.9 (19.4) |
| MVPA (min/week) *^*^* | 29.2 (9.1) | 27.2 (9.4) | 31.1 (8.4) |
| Step counts (steps per week) *^*^* | 6803 (1573.0) | 5609 (1019.3) | 7998 (1023.0) |
| Sedentary school time (min/week) | 1487.8 (40.7) | 1490.7 (53.1) | 1485.0 (22.9) |
| **Health Outcomes** |  |  |  |
| Body-mass-index (M+/- SD) | 15.7 (2.6) | 16.0 (2.7) | 15.5 (2.6) |
| Aerobic fitness in (*ml/kg/min)* | 19.8 (1.3) | 20.0 (1.5) | 19.6 (1) |
| Perceived stress *(total score out of 4)* | 1.8 (0.4) | 1.8 (0.4) | 1.8 (0.4) |
| Perceived psychological stress | 1.9(0.4) | 1.9 (0.4) | 1.9 (0.4) |
| Perceived physiological stress | 1.7(0.5) | 1.7 (0.5) | 1.8 (0.5) |
| *Note:^*^P < 0.05;* ^a^Sample of movement behaviours = 92 (IG = 46, CG = 46); IG = intervention group; CG = control group; SD = standard deviation; PA = physical activity; MVPA = moderate-to-vigorous physical activity; ^b^The subsample was consisted of 55 boys(59.8%) and 37 girls (40.2%) (IG – 28 and 18, CG – 23 and 19 boys and girls respectively). | | | |

## Mean Values of the IG and CG at Baseline and Post-test.

Table 2. Change of mean scores over time

| **Outcome** | **Mean (SD) at T1** | | **Mean (SD) at T2** | |
| --- | --- | --- | --- | --- |
|  | **IG** | **CG** | **IG** | **CG** |
| Mathematics | 80.8 (16.3) | 83.1 (12.3) | 68.9 (17.2) | 69.4 (19.1) |
| Reading^a^ | 82.2 (13.9) | 76.7 (10.5) | 88.0 (12.9) | 77.6 (13.0) |
| LPA | 282.1 (47.9) | 283.9 (19.4) | 280.3 (53.9) | 120.5 (36.3) |
| MVPA^a^ | 27.2 (9.4) | 31.1 (8.4) | 44.3 (9.9) | 24.3 (9.4) |
| Average MVPA^a^ | 5.4 (1.9) | 6.2 (1.7) | 8.9 (2.0) | 4.9 (1.9) |
| Step count^a^ | 5609.0 (1019.3) | 7998.5 (1023.0) | 7769.3 (1339.0) | 3942.9 (1046.3) |
| Sedentary school time | 1490.7 (53.1) | 1485.0 (22.9) | 1475.4 (59.4) | 1655.1 (40.8) |
| BMI | 282.1 (47.9) | 283.9 (19.4) | 15.1 (2.5) | 15.0 (3.0) |
| Aerobic fitness | 27.2 (9.4) | 31.1 (8.4) | 20.5 (1.4) | 19.8 (1.2) |
| Perceived stress | 5.4 (1.9) | 6.2 (1.7) | 1.6 (0.4) | 1.6 (0.4) |
| Perceived psychological stress | 5609.0 (1019.3) | 7998.5 (1023.0) | 1.7 (0.5) | 1.7 (0.4) |
| Perceived physiological stress | 1490.7 (53.1) | 1485.0 (22.9) | 1.5 (0.4) | 1.6 (0.4) |
| Note. ^1^Type III tests; LPA = light physical activity (min/week); MVPA = moderate-to-vigorous physical activity (min/week); BMI = body-mass-index; SD = standard deviation; df = degree of freedom; IG = intervention group; CG = waitlist control group; T2 = post-intervention assessment; ^a^ = adjusted for baseline differences. | | | | |

Figure 5. Mean Values of Academic Achievement Outcomes at T1 and T2

Figure 6. Mean Values of Physical Activity Levels at T1 and T2

Figure 7. Mean Values of Step Count and Sedentary School Time at T1 and T2

Figure 8. Mean Values of Health-related Outcomes at T1 and T2

Table 3. Results of the intervention effects and the moderation effects by gender (N = 220)

| **Outcome** | **Mean (SD) at T1** | | **Mean (SD) at T2** | | **Time×group^1^** | | **β [95% CI]** | **Time^1^** | | **Group^1^** | | **Time×group×Gender^1^** | |  |
| --- | --- | --- | --- | --- | --- | --- | --- | --- | --- | --- | --- | --- | --- | --- |
|  | **IG** | **CG** | **IG** | **CG** | ***F/χ*^2^ test (*df*)** | ***P* value** |  | ***F/χ*^2^ test (*df*)** | ***P* value** | ***F/χ*^2^ test (*df*)** | ***P* value** | ***F/χ*^2^ test (*df*)** | ***P* value** |  |
| Reading^a^  (Cohen’s *d*=0.5) | 82.2 (13.9) | 76.7 (10.5) | 88.0 (12.9) | 77.6 (13.0) | 15.8 (1,208) | **<.001** | 4.6 [1.5,7.7] | 33.0 (1,208) | <.001 | 32.5 (1,208) | <.001 | 0.1(1,208) | 0.752 |  |
| Mathematics  (Cohen’s *d*=0.1) | 80.8 (16.3) | 83.1 (12.3) | 68.9 (17.2) | 69.4 (19.1) | 0.7 (1,216) | 0.384 | 3.5 [-1.3,8.3] | 170.3 (1,216) | <.001 | 0.2 (1,216) | 0.657 | 0.9 (1,216) | 0.346 |  |
| LPA  (Cohen’s *d*=1.9) | 282.1 (47.9) | 283.9 (19.4) | 280.3 (53.9) | 120.5 (36.3) | 185.0 (1,88) | **<.001** | 141.4 [110.4172.3] | 170.4 (1,88) | <.001 | 160.3 (1,88) | <.001 | 4.4 (1,88) | **0.039**^b^ |  |
| MVPA^a^  (Cohen’s *d*=1.2) | 27.2 (9.4) | 31.1 (8.4) | 44.3 (9.9) | 24.3 (9.4) | 76.2 (1,85) | **<.001** | 22.4[15.1,29.7] | 12.9 (1,85) | <.001 | 36.9 (1,85) | <.001 | 0.7 (1,85) | 0.356 |  |
| Step count^a^  (Cohen’s *d*=2.571 | 5609.0 (1019.3) | 7998.5 (1023.0) | 7769.3 (1339.0) | 3942.9 (1046.3) | 357.2 (1,85) | **<.001** | 6051.1 [5186.9,6915.2] | 32.1 (1,85) | <.001 | 16.7 (1,85) | <.001 | 1.4 (1,85) | 0.244 |  |
| Sedentary school time  (Cohen’s *d*=1.9) | 1490.7 (53.1) | 1485.0 (22.9) | 1475.4 (59.4) | 1655.1 (40.8) | 202.1 (1,88) | **<.001** | -162.7 [-196.7, -128.8] | 123.1 (1,88) | <.001 | 155.0 (1,88) | <.001 | 4.6 (1,88) | **0.035**^c^ |  |
| BMI  (Cohen’s *d*=0.1) | 16.0 (2.7) | 15.5 (2.6) | 15.1 (2.5) | 15.0 (3.0) | 0.8 (1,216) | 0.380 | -0.2 [-0.8,0.5] | 23.0(1,216) | <.001 | 0.7(1,216) | 0.399 | 0.0 (1,216) | 0.848 |  |
| Aerobic fitness  (Cohen’s *d*=0.2) | 20.0 (1.5) | 19.6 (1) | 20.5 (1.4) | 19.8 (1.2) | 2.9 (1,216) | 0.088 | 0.2 [-0.4,0.7] | 12.3 (1,216) | <.001 | 9.9 (1,216) | .002 | 0.8 (1,216) | 0.365 |  |
| Perceived stress  (Cohen’s *d*=0.1) | 1.8 (0.4) | 1.8 (0.4) | 1.6 (0.4) | 1.6 (0.4) | 0.5 (1,216) | 0.499 | 0.0 [-0.1,0.2] | 58.1 (1,216) | <.001 | 0.8(1,216) | 0.364 | 0.0 (1,216) | 0.916 |  |
| Perceived psychological stress (Cohen’s *d*=0.1) | 1.9 (0.4) | 1.9 (0.4) | 1.7 (0.5) | 1.7 (0.4) | 0.7 (1,216) | 0.416 | 0.0 [-0.1,0.2] | 32.6 (1,216) | <.001 | 0.0(1,216) | 0.925 | 0.0 (1,216) | 0.869 |  |
| Perceived physiological stress (Cohen’s *d*=0.0) | 1.7 (0.5) | 1.8 (0.5) | 1.5 (0.4) | 1.6 (0.4) | 0.1 (1,216) | 0.809 | 0.0 [-0.1,0.2] | 41.61 (1,216) | <.001 | 2.7(1,216) | 0.100 | 0.1 (1,216) | 0.748 |  |
| Note. ^1^Type III tests; LPA = light physical activity (min/week); MVPA = moderate-to-vigorous physical activity (min/week); BMI = body-mass-index; SD = standard deviation; df = degree of freedom; IG = intervention group; CG = waitlist control group; T2 = post-intervention assessment; ^a^ = adjusted for baseline differences; D = Type III fixes effect test statistic; β = estimated intervention effect at T2; CI = confidence interval; IG, T2 and male students are the reference categories; ^b^:(β = 51.6, 95% CI [2.8,100.4]); ^c^:(β = -57.8, 95% CI [-111.4,-4.2). | | | | | | | | | | | | | | |

Table 4. Intervention effects – sensitivity analysis (N = 92)

| **Outcome** | **Mean (SD) at T2** | | **Time×group^1^** | | **Time^1^** | | **Group^1^** | | **Time×group×Gender^1^** | | **Gender^1^** | |
| --- | --- | --- | --- | --- | --- | --- | --- | --- | --- | --- | --- | --- |
|  | **IG** | **CG** | ***F/χ*^2^ test (*df*)** | ***P* value** | ***F/χ*^2^ test (*df*)** | ***P* value** | ***F/χ*^2^ test (*df*)** | ***P* value** | ***F/χ*^2^ test (*df*)** | ***P* value** | ***F/χ*^2^ test (*df*)** | ***P* value** |
| Mathematics | 71.78  (17.4) | 69.4 (19.1) | 0.670 (1,88) | 0.415 | 65.422 (1,88) | <.001 | 0.051 (1,88) | 0.821 | 0.889 (1,88) | 0.351 | 0.835 (1,88) | 0.363 |
| Reading^a^ | 88.8 (13.7) | 76.8 (13.8) | 3.892 (1,87) | 0.052 | 6.026 (1,87) | 0.016 | 17.677 (1,87) | <.001 | 2.854 (1,87) | 0.095 | 1.595 (1,87) | 0.210 |
| BMI | 15.1 (2.8) | 14.6 (2.5) | 1.089 (1,88) | 0.300 | 12.452 (1,88) | <.001 | 1.772 (1,88) | 0.187 | 0.809 (1,88) | 0.371 | 0.001 (1,88) | 0.977 |
| Aerobic fitness | 20.5 (1.5) | 20.0 (1.3) | 0.520 (1,88) | 0.473 | 5.578 (1,88) | 0.020 | 2.964 (1,88) | .089 | 2.273 (1,88) | 0.135 | 1.903 (1,88) | 0.171 |
| Perceived stress | 1.5 (0.3) | 1.6 (0.4) | 1.881 (1,88) | 0.174 | 29.186 (1,88) | <.001 | 0.148 (1,88) | 0.701 | 0.180 (1,88) | 0.672 | 5.502 (1,88) | 0.021 |
| Perceived psychological stress | 1.6 (0.4) | 1.6 (0.4) | 0.038 (1,88) | 0.846 | 26.895 (1,88) | <.001 | 0.086 (1,88) | 0.771 | 0.994 (1,88) | 0.322 | 4.379 (1,88) | 0.039 |
| Perceived physiological stress | 1.4 (0.4) | 1.6 (0.5) | 4.199 (1,88) | 0.43 | 13.257 (1,88) | <.001 | 0.813 (1,88) | 0.370 | 0.086 (1,88) | 0.770 | 4.089 (1,88) | 0.046 |
| Note. ^1^Type III tests; BMI = body-mass-index; SD = standard deviation; df = degree of freedom; IG = intervention group; CG = waitlist control group; T2 = post-intervention assessment; ^a^ = adjusted for baseline differences. | | | | | | | | | | | | |

## Supplementary file 4: Detailed information: Intervention fidelity.

Figure 5. Weekly Implementation of IcPAB by Class Teachers

Table 5. Fidelity of the intervention of each classroom

**Teacher ID: 1**

| Activity | Week1 | Week2 | Week3 | Week4 | Week5 | Week6 | Week7 | Week8 | Week9 | Week10 | Week11 | Week12 |
| --- | --- | --- | --- | --- | --- | --- | --- | --- | --- | --- | --- | --- |
| IcPAB_1 | 5 | 2 | 4 | 5 | 4 | 1 | 2 | 3 |  | 1 |  | 3 |
| IcPAB_2 |  | 1 |  | 1 | 1 | 2 |  | 1 | 3 | 2 | 1 |  |
| IcPAB_3 | 1 | 3 | 1 |  | 1 | 1 | 1 | 1 |  |  | 1 |  |
| IcPAB_4 | 1 | 2 |  | 1 |  |  |  | 1 | 3 |  | 3 | 1 |
| IcPAB_5 | 1 |  | 1 | 1 | 1 |  |  |  | 1 |  |  |  |
| IcPAB_6 | 1 | 2 |  | 1 | 1 |  |  | 2 | 1 | 1 | 1 |  |
| IcPAB_7 | 1 | 1 | 2 |  | 1 | 1 | 1 | 2 |  | 1 | 1 |  |
| IcPAB_8 | 1 | 1 | 2 | 1 | 2 |  | 1 |  |  | 1 | 2 | 1 |
| IcPAB_9 | 1 | 1 | 1 |  |  |  |  |  |  |  |  |  |
| IcPAB_10 | 1 |  |  | 1 | 2 | 1 |  | 3 | 2 | 2 | 1 | 1 |
| IcPAB_11 | 1 | 1 | 1 | 1 |  |  |  |  |  |  |  |  |
| IcPAB_12 |  | 1 | 1 |  |  |  | 1 | 1 |  |  |  |  |
| IcPAB_13 |  | 1 | 1 |  |  | 1 |  |  |  | 1 | 2 |  |
| IcPAB_14 |  | 2 | 2 |  |  |  | 1 |  | 1 |  |  | 1 |
| IcPAB_15 |  |  |  | 1 | 1 |  |  | 2 |  |  |  |  |
| IcPAB_16 | 1 |  |  | 1 | 1 | 2 |  | 1 |  | 1 | 1 | 1 |
| IcPAB_17 | 1 | 1 | 1 |  |  |  |  |  |  |  |  |  |
| IcPAB_18 | 1 | 2 |  | 1 | 1 |  |  | 3 | 1 | 1 |  | 1 |
| IcPAB_19 |  |  |  |  |  | 2 |  |  |  |  |  |  |
| IcPAB_20 |  | 2 |  | 1 |  | 1 |  |  |  |  | 2 | 1 |
| Other |  |  |  |  |  |  |  |  |  |  |  |  |
| Total | 17 | 23 | 17 | 16 | 16 | 12 | 7 | 20 | 12 | 11 | 15 | 10 |
| Average | 3.4 | 4.6 | 3.4 | 3.2 | 3.2 | 2.4 | 1.4 | 4 | 2.4 | 2.2 | 3 | 2 |

**Teacher ID: 2**

| Activity | Week1 | Week2 | Week3 | Week4 | Week5 | Week6 | Week7 | Week8 | Week9 | Week10 | Week11 | Week12 |
| --- | --- | --- | --- | --- | --- | --- | --- | --- | --- | --- | --- | --- |
| IcPAB_1 | 5 | 3 | 4 | 1 | 1 |  | 1 | 1 | 1 | 1 | 2 | 2 |
| IcPAB_2 | 1 |  |  | 1 |  |  |  | 1 |  | 1 | 1 |  |
| IcPAB_3 | 2 |  |  | 3 | 1 |  | 1 | 1 | 1 | 1 |  |  |
| IcPAB_4 |  | 1 |  | 1 | 1 | 1 |  | 1 |  |  |  | 1 |
| IcPAB_5 | 1 |  |  |  | 1 | 1 |  | 1 | 1 | 1 | 1 | 1 |
| IcPAB_6 | 2 |  | 1 | 1 |  |  | 2 | 1 | 1 |  |  | 1 |
| IcPAB_7 |  |  |  |  | 2 |  | 1 | 1 |  | 1 | 1 |  |
| IcPAB_8 | 1 | 2 |  | 1 |  | 1 |  | 1 | 1 | 1 | 1 | 2 |
| IcPAB_9 |  |  | 3 | 1 | 2 |  |  | 2 | 2 | 1 | 1 |  |
| IcPAB_10 |  |  | 1 |  | 1 |  |  | 1 | 2 |  | 1 | 1 |
| IcPAB_11 | 1 |  |  | 1 |  |  | 1 | 2 | 1 | 1 |  | 1 |
| IcPAB_12 |  |  |  | 1 |  | 1 |  | 1 |  |  | 1 | 1 |
| IcPAB_13 |  | 1 | 1 | 1 |  |  |  | 1 |  |  |  |  |
| IcPAB_14 |  |  |  | 2 |  |  |  |  |  | 1 | 1 | 1 |
| IcPAB_15 |  |  |  |  | 1 |  |  |  |  | 1 |  |  |
| IcPAB_16 |  |  |  |  |  |  | 2 |  | 1 |  | 1 |  |
| IcPAB_17 |  | 1 |  | 1 |  | 3 |  |  | 1 |  |  | 1 |
| IcPAB_18 |  |  | 1 |  | 2 |  | 2 |  | 1 | 1 |  |  |
| IcPAB_19 |  | 1 |  | 1 | 2 |  |  | 1 |  |  |  |  |
| IcPAB_20 |  |  |  |  | 3 |  |  | 1 |  |  | 1 |  |
| Other |  |  |  |  |  |  |  |  |  |  |  |  |
| Total | 13 | 9 | 11 | 16 | 17 | 7 | 10 | 17 | 13 | 11 | 12 | 12 |
| Average | 2.6 | 1.8 | 2.2 | 3.2 | 3.4 | 1.4 | 2 | 3.4 | 2.6 | 2.2 | 2.4 | 2.4 |

**Teacher ID: 3**

| Activity | Week1 | Week2 | Week3 | Week4 | Week5 | Week6 | Week7 | Week8 | Week9 | Week10 | Week11 | Week12 |
| --- | --- | --- | --- | --- | --- | --- | --- | --- | --- | --- | --- | --- |
| IcPAB_1 | 2 | 3 | 4 | 2 | 3 | 1 | 3 |  | 1 | 1 | 2 | 2 |
| IcPAB_2 |  |  | 1 | 1 | 1 | 2 |  | 1 | 1 | 2 | 1 |  |
| IcPAB_3 | 2 |  |  |  | 2 | 1 | 1 | 2 | 1 | 2 | 1 |  |
| IcPAB_4 |  | 1 |  | 2 |  | 2 | 2 |  | 2 |  | 1 | 1 |
| IcPAB_5 | 1 |  |  |  |  |  | 1 |  |  | 1 |  | 1 |
| IcPAB_6 | 2 |  | 1 | 2 | 1 | 1 |  | 1 | 1 | 1 | 1 | 1 |
| IcPAB_7 |  |  |  | 1 | 1 |  | 1 | 1 |  | 2 |  |  |
| IcPAB_8 | 1 | 2 |  | 1 |  |  |  | 1 | 3 |  | 1 | 1 |
| IcPAB_9 |  |  | 2 |  |  | 1 | 1 |  | 1 | 1 |  | 1 |
| IcPAB_10 |  |  | 1 |  | 1 |  | 2 |  |  |  |  |  |
| IcPAB_11 | 1 |  |  | 1 | 1 |  |  | 1 |  |  | 1 | 1 |
| IcPAB_12 |  |  |  |  | 1 | 1 |  |  |  |  |  |  |
| IcPAB_13 |  | 1 | 1 | 1 | 1 | 1 | 1 |  | 2 | 1 |  | 1 |
| IcPAB_14 |  |  |  | 1 |  | 1 |  | 1 | 1 |  | 1 |  |
| IcPAB_15 |  |  |  | 1 | 1 |  |  |  |  | 1 | 1 |  |
| IcPAB_16 |  |  |  |  | 1 | 1 | 1 |  | 1 | 1 |  | 1 |
| IcPAB_17 |  | 1 |  | 1 |  |  |  | 1 |  |  |  |  |
| IcPAB_18 |  |  | 1 | 1 |  | 1 | 1 |  | 2 |  |  | 1 |
| IcPAB_19 |  | 1 |  |  | 1 |  |  | 1 | 1 |  |  |  |
| IcPAB_20 |  |  |  | 1 | 1 | 1 | 1 | 1 |  | 1 |  |  |
| Other |  |  |  |  | 1 |  |  |  |  |  |  |  |
| Total | 9 | 9 | 11 | 16 | 17 | 14 | 15 | 11 | 17 | 14 | 10 | 11 |
| Average | 1.8 | 1.8 | 2.2 | 3.2 | 3.4 | 2.8 | 3 | 2.2 | 3.4 | 2.8 | 2 | 2.2 |

## Supplementary file 5: Process evaluation results

Table 6. Feedback: IcPAB's process

| **Process evaluation statement (students)** | Very strongly agreed | Agreed | Neutral | Did not agree | Did not agree at all |
| --- | --- | --- | --- | --- | --- |
| I happily engaged in the IcPAB activities | 89 (98.9%) | 1 (1.1%) | - | - | - |
| I like to IcPAB in future | 83 (92.2%) | 5 (5.6%) | 2 (2.2%) | - | - |
| I felt happier than before | 78 (86.7%) | 11 (12.2%) | 1 (1.1%) | - | - |
| I could easily do IcPAB | 66 (73.3%) | 19 (21.1%) | 1 (1.1%) | 1 (1.1%) | 3 (3.3%) |
| I easily understood instructions | 80 (88.9%) | 6 (6.7%) | 3 (3.3%) | 1 (1.1%) | - |
| I had enough space for IcPAB | 63 (70%) | 14 (15.6%) | 2 (2.2%) | 3 (3.3%) | 8 (8.9%) |
| Allocated time was enough | 42 (46.7%) | 6 (6.7%) | 5 (5.6%) | - | 37 (41.1%) |
| I did enough IcPAB everyday | 41 (45.6%) | 6 (6.7%) | 5 (5.6%) | 4 (4.4) | 34 (37.8) |
| The speed of IcPAB was enough | 72 (80%) | 3 (3.3%) | 5 (5.6%) | 2 (2.2%) | 8 (8.9%) |
| **Process evaluation statement (teachers)** | Very strongly agreed | Agreed | Neutral | Did not agree | Did not agree at all |
| IcPAB fitted well to the routine | 3 (100%) | - | - | - | - |
| IcPAB were easy to adapt | 2 (66.7%) | 1 (33.3%) | - | - | - |
| IcPAB created many additional work | - | - | 2 (66.7%) | - | 1 (33.3%) |
| IcPAB was not flexible | - | - | 1 (33.3%) | 1 (33.3%) | 1 (33.3%) |
| IcPAB had much to do | - | - | 2 (66.7%) | - | 1 (33.3%) |
| Had to allocate too long time | - | - | - | - | 3 (100%) |
| I will use IcPAB in future | 3 (100%) | - | - | - | - |
| I recommend IcPAB to others | 3 (100%) | - | - | - | - |
